# MRI Compatible, Customizable, and 3D-Printable Microdrive for Neuroscience Research

**DOI:** 10.1523/ENEURO.0495-20.2021

**Published:** 2021-03-05

**Authors:** Eunha Baeg, Raymond Doudlah, Robert Swader, Hyowon Lee, Minjun Han, Seong-Gi Kim, Ari Rosenberg, Byounghoon Kim

**Affiliations:** ^1^Center for Neuroscience Imaging Research, Institute for Basic Science, Suwon, Republic of Korea 16060; ^2^Department of Biomedical Engineering, Sungkyunkwan University, Suwon, Republic of Korea 16419; ^3^Department of Neuroscience, School of Medicine and Public Health, University of Wisconsin–Madison, Madison, WI 53705; ^4^Morgridge Institute for Research, Madison, WI 53705; ^5^System Design Engineering, University of Waterloo, Waterloo, Ontario, Canada N2L 3G1

**Keywords:** 3D printing, effective connectivity, electrical microstimulation, electrophysiology, microdrive, MRI compatible

## Abstract

The effective connectivity of brain networks can be assessed using functional magnetic resonance imaging (fMRI) to quantify the effects of local electrical microstimulation (EM) on distributed neuronal activity. The delivery of EM to specific brain regions, particularly with layer specificity, requires MRI compatible equipment that provides fine control of a stimulating electrode’s position within the brain while minimizing imaging artifacts. To this end, we developed a microdrive made entirely of MRI compatible materials. The microdrive uses an integrated penetration grid to guide electrodes and relies on a microdrilling technique to eliminate the need for large craniotomies, further reducing implant maintenance and image distortions. The penetration grid additionally serves as a built-in MRI marker, providing a visible fiducial reference for estimating probe trajectories. Following the initial implant procedure, these features allow for multiple electrodes to be inserted, removed, and repositioned with minimal effort, using a screw-type actuator. To validate the design of the microdrive, we conducted an EM-coupled fMRI study with a male macaque monkey. The results verified that the microdrive can be used to deliver EM during MRI procedures with minimal imaging artifacts, even within a 7 Tesla (7T) environment. Future applications of the microdrive include neuronal recordings and targeted drug delivery. We provide computer aided design (CAD) templates and a parts list for modifying and fabricating the microdrive for specific research needs. These designs provide a convenient, cost-effective approach to fabricating MRI compatible microdrives for neuroscience research.

## Significance Statement

We provide designs for a customizable, magnetic resonance imaging (MRI) compatible microdrive capable of positioning various types of probes (e.g., stimulating electrodes, recording electrodes, drug cannulae, or optogenetic fibers) within the brain. The design integrates a cranial implant, penetration grid for guiding probes, and a microdrive body assembly with actuators. A microdrilling technique, which helps reduce implant maintenance and potential imaging artifacts, is described for introducing probes into the brain. Our open-source designs allow for the customization and fabrication of microdrive components to meet the unique demands of specific research projects and various animal models. Microdrives based on these designs can fulfill a variety of research needs within the neuroscience community related to electrical microstimulation, neuronal recording, and local drug delivery.

## Introduction

Hemodynamic responses measured using functional magnetic resonance imaging (fMRI) can be used to study the organization of brain networks (Ogawa and Lee, 1990; Kourtzi et al., 2003; Logothetis, 2003). In particular, fMRI signals evoked by local electrical microstimulation (EM) can reveal the effective connectivity of different brain areas (Tolias et al., 2005; Logothetis et al., 2010; Premereur et al., 2015; Dromme et al., 2016; Duffau, 2020; Premereur and Janssen, 2020). An MRI compatible microdrive capable of positioning various types of probes (e.g., stimulating electrodes, recording electrodes, drug cannulae, or optogenetic fibers) can facilitate a variety of fMRI-based studies. Indeed, incorporating neuromodulation approaches, such as EM and pharmacological methods, into non-human primate neuroimaging studies was recently identified as a five-year goal of “unprecedented value” (Milham et al., 2020).

Currently, relatively few commercially or academically available microdrives can support this goal (Table 1), and several factors limit discovery. First, long travel distances may be required to reach a desired brain region. For example, >30 mm of travel is required to reach some ventral brain regions in macaques. However, only a few available MRI compatible microdrives can travel such long distances. Second, a scanner’s bore size can preclude the use of stereotactic manipulators because of the length of the electrode holders. Third, electrode holders can amplify mechanical vibrations from the scanner, resulting in tissue and/or probe damage. Frameless, skull-mounted microdrives provide effective solutions to problems of bore size and mechanical vibrations but generally have limited travel distance (Wilson et al., 2003; Greenberg and Wilson, 2004). To reduce duplicated efforts associated with groups developing their own microdrives *de novo* (Moeller et al., 2008; McMahon et al., 2014) and facilitate discovery, we designed a novel MRI compatible microdrive that can be customized to support a wide range of experimental needs.

**Table 1:** Example MRI compatible microdrives

Availability	Manufacturer/source	Construction style	Actuating mechanism	Electrode travel distance	Craniotomy required
Commercial	NeuroNexus, Inc. (MRI-compatible matrix array)	Stand-alone	N/A	Fixed length (max 15 mm)	Yes
	FHC, Inc. (NeuroPace)	Stand-alone	N/A	Fixed length based on electrode	No
	NaN Instruments, Inc. (NAN MRI drive)	Tower style	Motorized screw type	Max: 120 mm	Yes
Academic	Kern et al. (2008)	Stand-alone	Ultrasonic actuator	Max: 50 mm	Yes
	Grahn et al. (2016)	Frame-based stereotactic system	Manual screw type	Max: 12 mm	Yes
	Dotson et al. (2017)	Stand-alone	Manual screw type	Max: 20 mm	Yes
	Sudhakar et al. (2019)	Stand-alone	N/A	Fixed length based on electrode	Yes

Here, we present a frameless, skull-mounted MRI compatible microdrive that encapsulates a cranial implant, penetration grid, and actuating mechanism capable of positioning various types of probes over long travel distances. To minimize imaging artifacts, all components are MRI compatible. The penetration grid used in conjunction with a contrast agent is visible in structural scans, providing a fiducial reference for estimating penetration trajectories (Kalwani et al., 2009; Dubowitz and Scadeng, 2011; Glud et al., 2017). The penetration grid further serves as a guide for a microdrilling technique that replaces conventional craniotomies with small holes through which probes are introduced into the brain (Rosenberg et al., 2013; Laurens et al., 2016; Chang et al., 2020). Compared with conventional craniotomies and chambers, this technique reduces potential artifacts caused by air-filled spaces, the need for periodic debridement of granulation tissue, and potential deformation of the underlying neuronal tissue (Wilson et al., 2005; Spitler and Gothard, 2008; Premereur and Janssen, 2020).

We tested the microdrive by using it to introduce a stimulating electrode into the striatum of a macaque monkey and delivered EM during an fMRI session in a 7 Tesla (7T) scanner. Cerebral blood volume (CBV) with monocrystalline iron oxide nanoparticles (MIONs) was used to measure the effects of the delivered EM (Vanduffel et al., 2001; Kim et al., 2013). We provide computer aided design (CAD) files to facilitate modification and fabrication (https://osf.io/tnpmk; RRID:SCR_019247), such as adding/removing actuators and scaling components to accommodate different animal models. We anticipate that this cost-effective, customizable microdrive can support a wide range of neuroscience research requiring fine probe positioning.

## Materials and Methods

### Microdrive architecture and design

**Figure 1.**
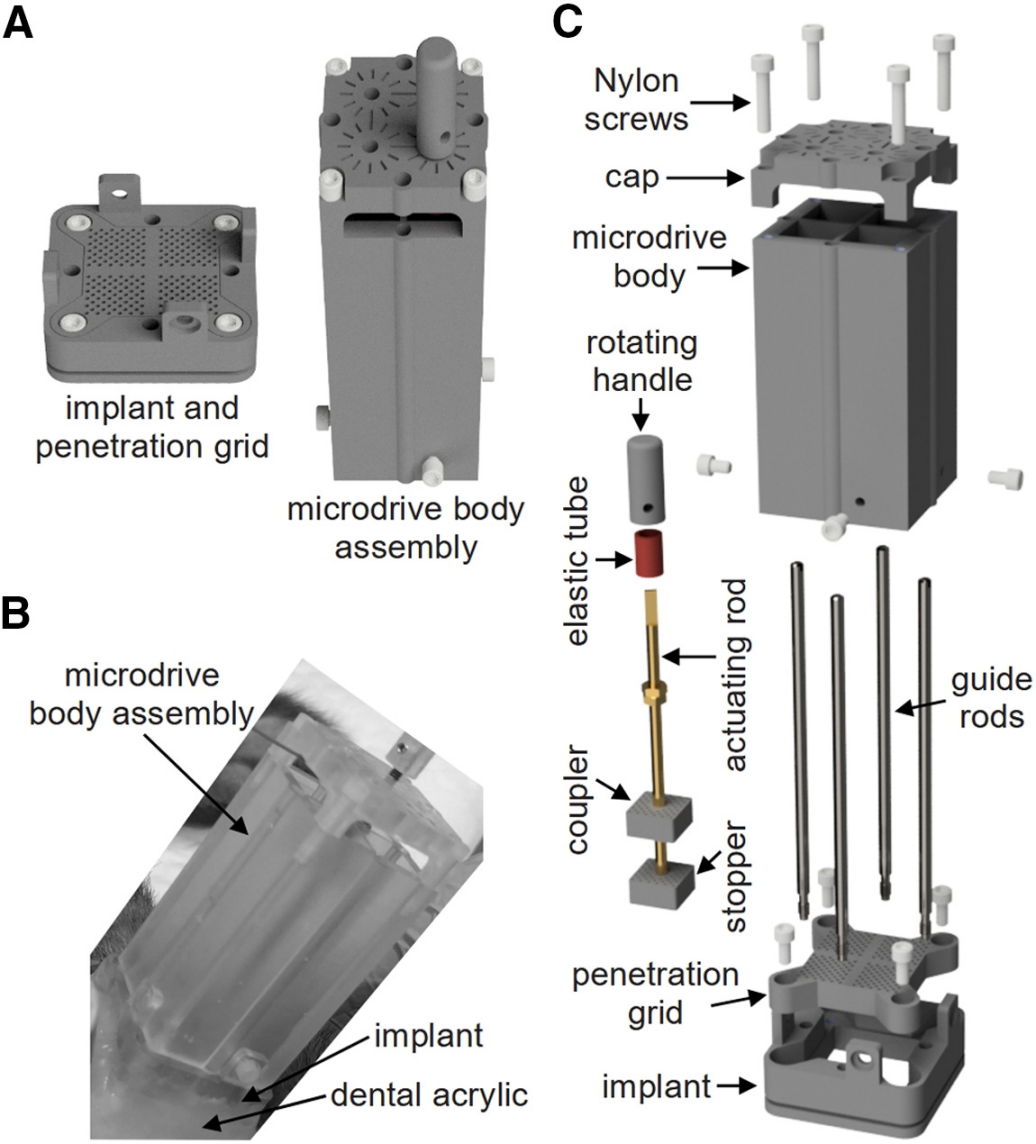
Overview of the microdrive design. ***A***, 3D rendering of the two major components of the microdrive: (1) implant and penetration grid (left) and (2) microdrive body assembly (right). ***B***, Picture of a 3D**-**printed microdrive body assembly installed on an implant that is fixed to a layer of dental acrylic over the cranium of a macaque monkey. ***C***, Exploded view of the microdrive to visualize the components and their assembly. All components are described in detail in the following sections and figures. CAD files and a parts list for all components are available for download (https://osf.io/tnpmk).

The microdrive consists of two major components: (1) the implant and penetration grid; and (2) the microdrive body assembly (Fig. 1*A*). The implant is fixed to the cranium and serves as an anchor frame for attaching the penetration grid and microdrive body assembly (Fig. 1*B*). The penetration grid provides a coordinate system for planning trajectories to reach specific brain areas and guides microdrilling for the insertion of guide tubes which protect the probes (e.g., stimulating electrodes, recording electrodes, drug cannulae, optogenetic fibers, etc.). In the presented design, the microdrive body assembly contains four chambers which align with the four quadrants of the penetration grid. Each chamber houses an individual actuator that independently controls a single probe (Fig. 1*C*).

CAD files for all parts and assemblies were created using SolidWorks (Dassault Systèmes, SolidWorks Corporation). A Viper si2 SLA System (3D Systems) with 0.0025 mm vertical resolution and 0.075 mm beam diameter was used for 3D printing. All 3D**-**printed components were made of Accura 60 photopolymer resin (3D Systems), which was selected for its chemical resistance and mechanical durability (tensile strength, 5868 MPa; tensile modulus, 2600–3100 MPa). Where necessary, 3D**-**printed parts were manually threaded with a standard 2–56 tap (56 threads per inch with a pitch of 453 μm) with the aid of a manual mill to maintain alignment. The actuating rod and guide rods (Fig. 1*C*) were not 3D-printed and required minor machining (as discussed in the following two sections).

#### Implant and penetration grid

**Figure 2.**
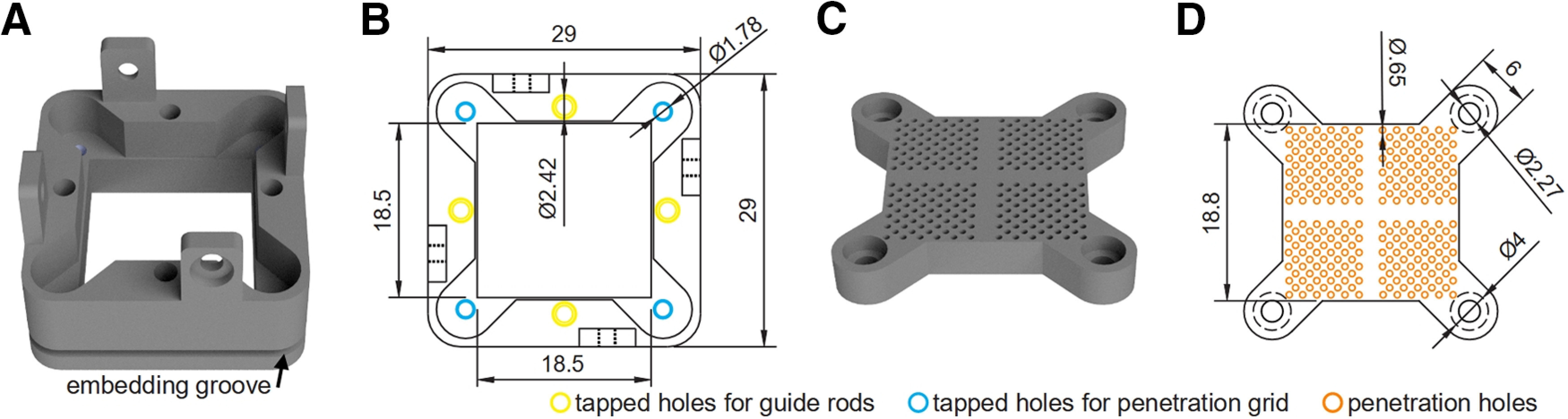
Implant and penetration grid. ***A***, 3D rendering of the implant. To help secure the implant to the cranium, the outer surface includes a groove (black arrow) which provides an embedding space for dental acrylic. Four vertical tongues are used to secure the microdrive body to the implant with Nylon screws. The cutout houses the penetration grid. ***B***, Top-view line drawing of the implant with dimensions. Tapped holes on each edge secure the guide rods (yellow circles). ***C***, 3D rendering of the penetration grid. ***D***, Top-view line drawing of the penetration grid with dimensions. The penetration grid is secured to the implant using Nylon screws (blue circles in ***B***). Each quadrant contains penetration holes (orange circles) which are used to estimate probe trajectories and guide microdrilling. Dimensions are in millimeters.

The implant is a square frame (29 × 29 mm) with four vertical tongues for securing the microdrive body assembly (Fig. 2*A*) and a threaded hole on each edge for securing guide rods (Fig. 2*B*, yellow circles). The guide rods are used to align the microdrive body assembly and implant during installation and removal, thereby preventing damage to the guide tubes and/or probes (Fig. 1*C*; also see Fig. 6*D*). These rods were made of MRI compatible 3/32” grade 5 titanium alloy and were turned on a lathe. To reduce image distortion, the guide rods can be unscrewed and removed after the microdrive body assembly has been secured to the implant. The outer surface of the implant contains a groove (Fig. 2*A*, black arrow) which serves as an embedding space when securing the implant to the cranium with dental acrylic (described below).

The main portion of the penetration grid is square in shape with anchor points in each corner that fit into the cutout of the implant (Fig. 2*C*,*D*). It is secured to the implant using four Nylon screws (Fig. 2*B*, blue circles). The grid has four quadrants, each with 61 penetration holes (ϕ0.65 mm; Fig. 2*D*, orange circles), providing a coordinate system for planning trajectories and guiding microdrilling for the insertion of guide tubes and probes. The location, number, and size of the holes can be modified based on experimental needs.

#### Microdrive body assembly

**Figure 3.**
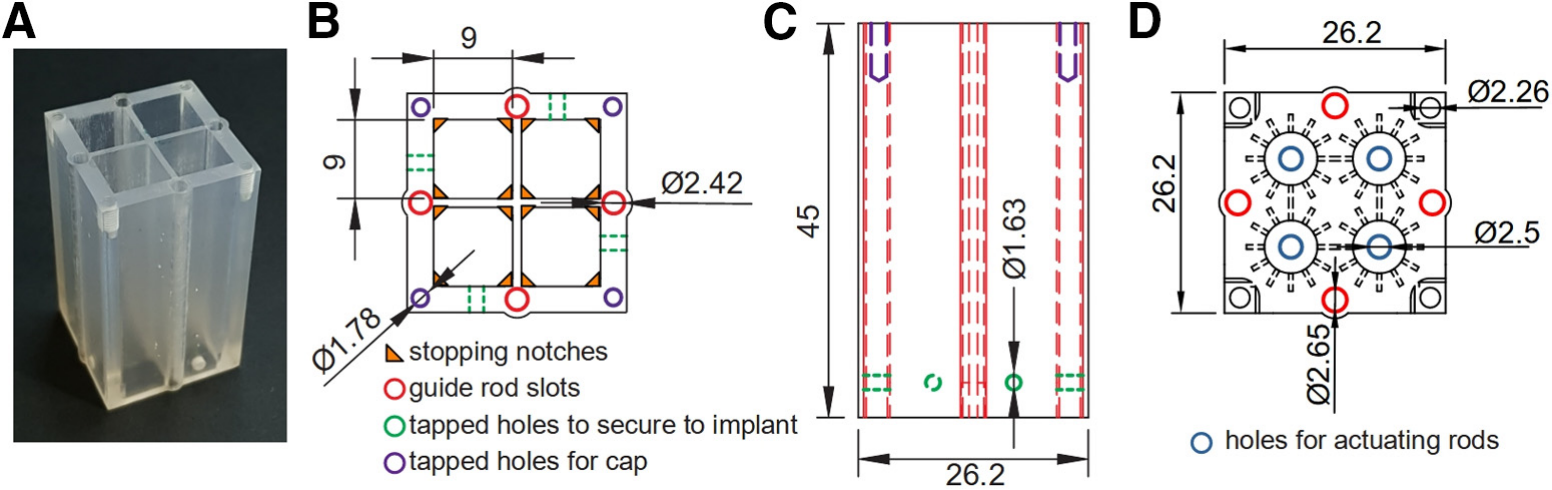
Microdrive body and cap. ***A***, Picture of a 3D**-**printed microdrive body with four chambers. Each chamber can house an independently controlled probe. ***B***, Top-view line drawing of the microdrive body with dimensions. Slots on each edge (red circles) fit over the guide rods to align the microdrive body with the implant and penetration grid. ***C***, Side-view line drawing of the microdrive body with dimensions. Red lines show the guide rod slots. The microdrive body secures to the implant via tapped holes on the bottom of each face (green lines/circles in ***B***, ***C***). ***D***, Top-view line drawing of the microdrive cap with dimensions. When the cap is installed, the ends of the actuating rods slide through holes in the cap (blue circles), exposing enough of the rods to secure rotating handles. The marks extending radially out from the actuating rod holes are indices for tracking the rotation of the actuating rods. The microdrive cap secures to the microdrive body via tapped holes (purple circles/lines in ***B***, ***C***) using Nylon screws. Dimensions are in millimeters.

**Figure 4.**
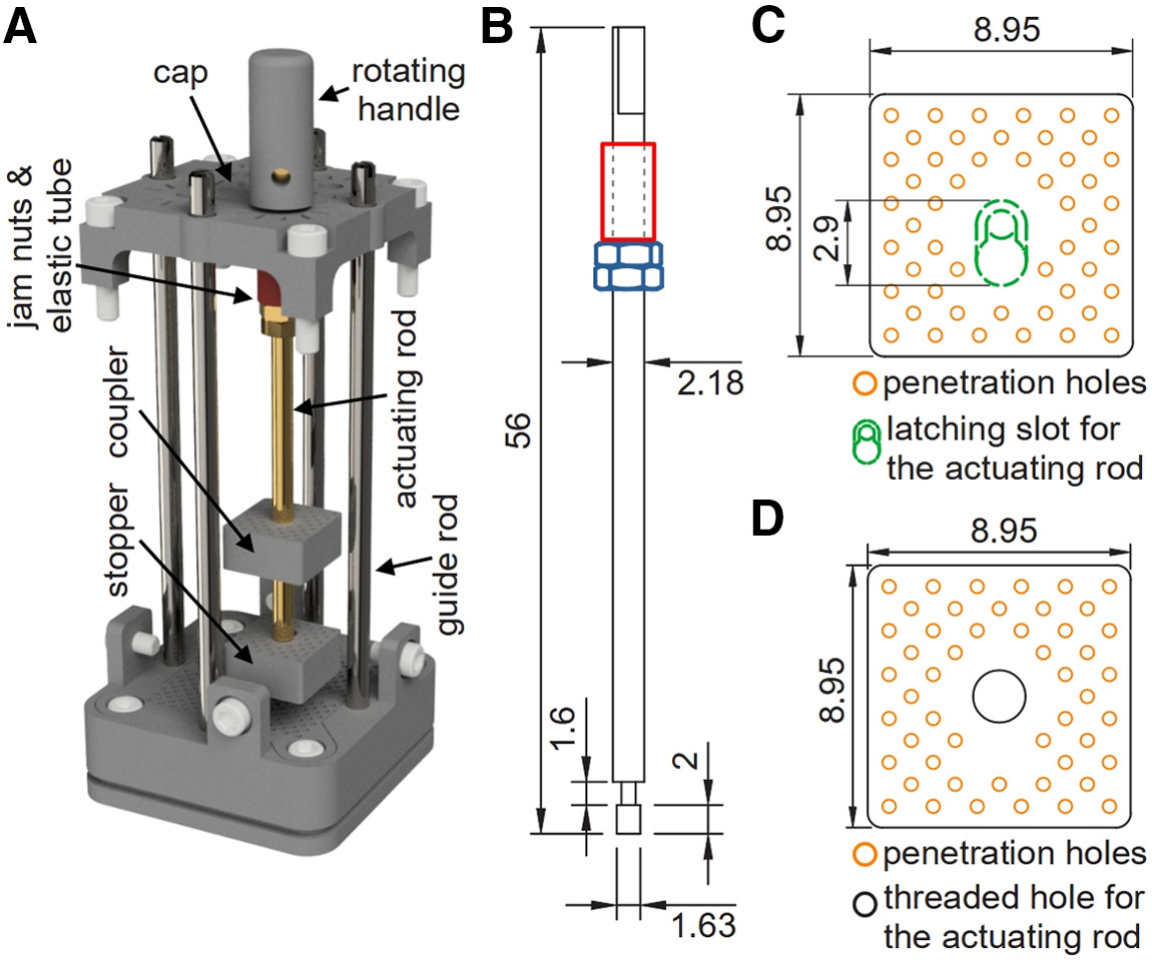
Actuating mechanism. ***A***, 3D rendering of the actuating mechanism, including the actuating rod, stopper, and coupler within the microdrive body (hidden). ***B***, Side-view line drawing of the actuating rod with dimensions. Counterclockwise rotation of the actuating rod advances the probe via the coupler. Jam nuts (in blue) provide an adjustable base to support an elastic tube (in red) which maintains tension between the jam nuts and the microdrive cap to ensure that the actuating mechanism does not inadvertently slide up the microdrive body chamber. The rotating handle attaches to the beveled end of the actuating rod (top). ***C***, Top-view line drawing of the stopper with dimensions. The latching slot (green dashed outline) attaches to the scalloped end of the actuating rod (***B***, bottom), securing the rod during rotations. Penetration holes (orange circles) align with corresponding holes in the coupler and penetration grid. ***D***, Top-view line drawing of the coupler with dimensions. The coupler screws onto the actuating rod via a threaded hole (black circle). This creates a screw and nut mechanism that controls the position of the coupler (and attached probe) by rotating the actuating rod. Penetration holes (orange circles) align with corresponding holes in the stopper and penetration grid (Fig. 2*D*). These holes ensure the positioning and alignment of the guide tube and probe during assembly so that the probe follows the intended trajectory. Dimensions are in millimeters.

The microdrive body assembly consists of two main components: (1) the microdrive body and cap; and (2) the actuating mechanism. In the presented design, the microdrive body contains four chambers (Fig. 3*A*), each of which can house a single actuating mechanism. The chambers are rectangular (9 × 9 × 45 mm) and align with the four quadrants of the penetration grid (Figs. 2*C*,*D*, 3*B*), providing positioning access for probes over a 353 mm^2^ area. The bottom corners of each chamber have small stopping notches on which the stopper of the actuating mechanism sits (Fig. 3*B*, orange triangles). This provides a base of support for the actuating mechanism and prevents it from sliding out the bottom of the chamber. Each face of the microdrive body contains a slot along its length for a guide rod (Fig. 3*B*, red circles, *C*, red lines). These rods align the microdrive body assembly with the penetration grid and implant to reduce the risk of damaging guide tubes or probes when installing/removing the microdrive body assembly onto/from the implant (see Fig. 6*D*). Nylon screws are used to secure the microdrive body to the implant via tapped holes on each face of the body (Fig. 3*B*, green lines, *C*, green circles and lines) which align with the tongues on the implant (Fig. 2*A*,*B*). The microdrive cap (Figs. 1*C*, 3*D*, 4*A*) holds and aligns the actuating mechanisms within the chambers of the microdrive body (Figs. 3*D*, blue circles, 4*A*). The cap also has measurement indicators for tracking the rotation of the actuating rods (Fig. 3*D*, marks extending radially from each actuating rod hole). The top of the microdrive body contains tapped holes in each corner for securing the cap with Nylon screws (Fig. 3*B*, purple circles, *C*, purple lines).

The actuating mechanism, which raises and lowers the probe, consists of three parts: (1) a threaded actuating rod; (2) stopper; and (3) coupler (Fig. 4*A*). The actuating rod has a scalloped end (Fig. 4*B*, bottom) that inserts into a latching slot in the center of the stopper (Fig. 4*C*). The stopper is square and fits snuggly within a chamber of the microdrive body. When an actuating mechanism is assembled and loaded into a chamber, the stopper sits on stopping notches at the bottom of the chamber to prevent the actuating mechanism from sliding out through the bottom of the microdrive body (Fig. 3*B*, orange triangles; also see Fig. 6*C*). The coupler fits within the chamber above the stopper and additionally threads onto the actuating rod to control the position of an attached probe (Fig. 4*D*). Because the coupler fits squarely within the chamber, rotating the actuating rod does not rotate the coupler. Instead, the rotation is converted into vertical translation via a screw and nut mechanism. The stopper provides an anchor point for the actuating rod and coupler that allows the actuating rod to freely rotate because of the latching slot (Fig. 4*A*). In the presented design, the actuating rod and coupler are threaded for 56 threads per inch, which lowers the coupler and attached probe 453 μm for each full counterclockwise turn of the actuating rod (clockwise rotations raise the coupler and probe). A rotating handle (Fig. 4*A*) attaches to the beveled end of the actuating rod (Fig. 4*B*, top) to assist with manual rotations. To prevent unwanted vertical translations of the actuating mechanism within the microdrive body chamber, two brass jam nuts (Fig. 4*B*, in blue) are used as an adjustable base to support an elastic tube (Fig. 4*B*, in red), which sits between the nuts and the microdrive cap (Fig. 4*A*). This structure mimics a compressed spring that creates downward pressure on the actuating mechanism from the microdrive cap, such that the stopper remains pressed against the stopping notches. Thus, the stopper and stopping notches prevent unwanted downward translations, while the brass nuts, elastic tube, and microdrive cap prevent unwanted upward translations. We made the actuating rods from an MRI compatible 2–56 threaded brass rod. The scalloped and beveled ends were manually machined.

Penetration holes in the stopper and coupler (Fig. 4*C*,*D*, orange circles) align with corresponding holes in the penetration grid. This alignment defines the penetration trajectory of an attached probe. The number, size, and location of these holes can be modified based on experimental needs. In the presented design, the microdrive can be used to individually control up to four probes simultaneously, one in each chamber. The total travel distance of a probe is determined by the length of the microdrive body and actuating rod (39 mm in this design).

### Cranial implant

**Figure 5.**
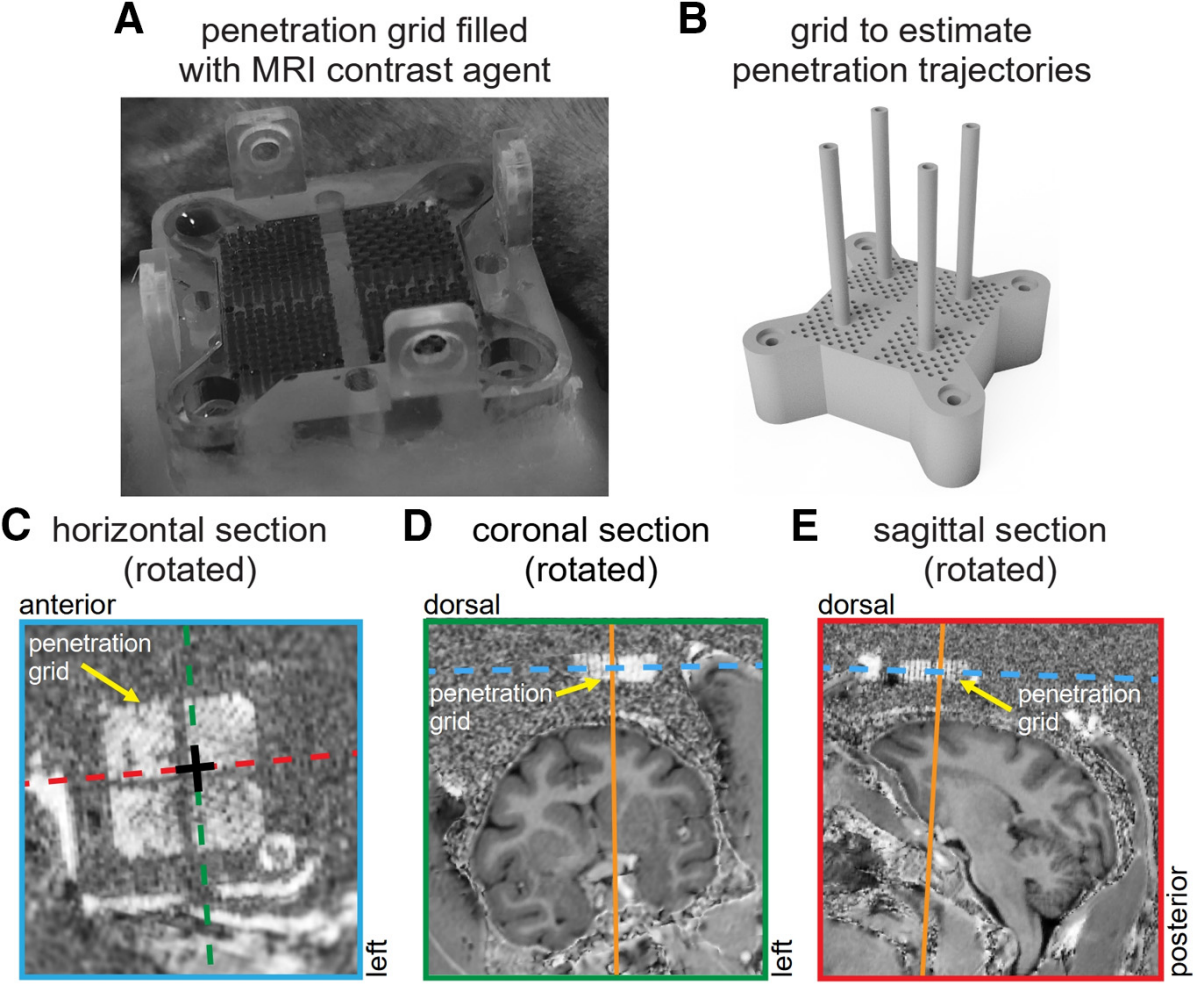
Estimating trajectories using the penetration grid. ***A***, Picture of the penetration grid secured to the implant and filled with an MRI contrast agent (povidone-iodine ointment). ***B***, 3D rendering of a specialized grid for making precise estimates of penetration trajectories. This grid has the same pattern of penetration holes as the standard penetration grid (***A***), but is thicker (10 mm, compared to 5 mm) and has four hollow pillars which are perpendicular to the grid surface for filling with an MRI contrast agent. These features can aid in the estimation of penetration trajectories. ***C****–****E***, Rotated MRI sections aligned relative to the horizontal plane of the penetration grid shown in ***A***. ***C***, Rotated horizontal MRI section providing a top-down view of the penetration grid. The four quadrants of the grid are visible and the penetration hole used in testing the microdrive is marked (black crosshair) along with the rotated coronal (green dashed line) and rotated sagittal (red dashed line) MRI sections. ***D***, Rotated coronal MRI section aligned relative to the horizontal plane defined by the penetration grid (blue dashed line), showing the estimated penetration trajectory (orange line). Note that the penetration trajectory is perpendicular to the horizontal plane of the penetration grid. ***E***, Same as ***D*** but for the rotated sagittal MRI section.

**Figure 6.**
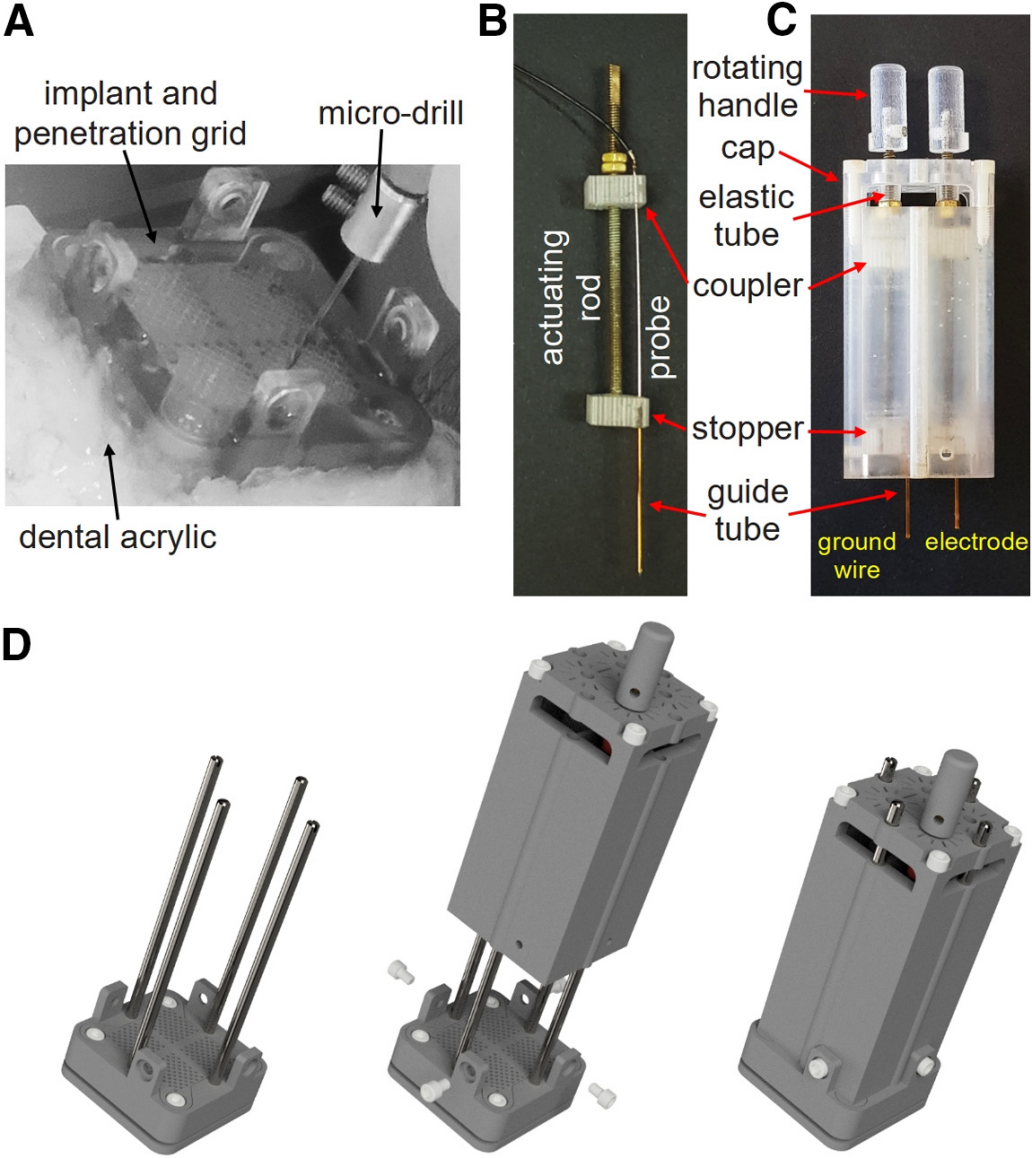
Preparing the microdrive body assembly and installing it on the implant. ***A***, Microdrilling through the penetration grid. The implant is secured to an acrylic layer over the cranium. The penetration grid is installed and a micro-drill is used to prepare the penetration site. ***B***, Prepared actuating mechanism. Here, a silica guide tube is secured to the stopper and a probe is secured to the coupler. ***C***, Prepared microdrive body assembly. Two actuating mechanisms are loaded in two separate chambers of the microdrive body. One actuating mechanism positions the ground wire (left) and the other positions the stimulating electrode (right). The microdrive cap is secured to the microdrive body and rotating handles are attached to the actuating rods. ***D***, 3D rendering of the installation of the microdrive body assembly onto the implant. Left, Four guide rods are installed on the implant. Middle, The prepared microdrive body assembly slides onto the four guide rods. Right, The microdrive body assembly is slowly lowered down onto the implant. Once in place, the microdrive body assembly is secured with four Nylon screws and the guide rods can be removed.

The implant serves as an anchoring point for the microdrive, so it needs to be secured to the cranium. The implant can be secured directly to the cranium with dental acrylic or to a layer of dental acrylic over the cranium (Figs. 1*B*, 5*A*; also see Fig. 6*A*). An advantage of affixing the implant to an acrylic layer, as opposed to the cranium directly, is that it can be readily repositioned within the bounds of the acrylic layer. The outer surface of the implant contains a groove which serves as an embedding space for the dental acrylic (Fig. 2*A*, black arrow). Once the acrylic hardens, thus securing the implant, the implant can be protected using a cover that attaches with four Nylon screws (not shown here but included with the CAD files).

### Estimating the penetration trajectory using a fiducial MRI marker

Estimating the trajectory and confirming the position of a probe within the brain is crucial to the success of an experiment but can be challenging without a stereotactic frame. Even with a stereotactic device, fine measurements of the distance and angle between the penetration point and target area can be difficult and imprecise. Using the holes in the penetration grid as a visible fiducial MRI marker helps mitigate this problem (Fig. 5). Specifically, the penetration grid can be filled with a contrast agent such as povidone-iodine ointment (Fig. 5*A*), making it visible in the MRI. In some cases, such as targeting small or deep structures, particularly precise estimates of the probe trajectory may be required. Such estimates can be made using a specialized grid (Fig. 5*B*). This grid has the same shape and guide hole configuration as the penetration grid but is thicker (10 vs 5 mm) and contains four hollow pillars that are perpendicular to the grid surface. By filling this grid and the pillars with a contrast agent, these features can aid in making more precise estimates of penetration trajectories. As illustrated in Figure 5*C–E*, the fiducial marker provided by the standard penetration grid (Fig. 5*A*) can also be used to estimate the probe’s trajectory (Kalwani et al., 2009; Dubowitz and Scadeng, 2011; Glud et al., 2017). Here, the structural MRIs were rotated and aligned to the horizontal plane of the penetration grid using 3DSlicer (Kikinis et al., 2014). In this way, penetration trajectories can be determined in a simple coordinate system defined by the penetration grid. As shown in Results, we tested the microdrive by delivering EM to the striatum during an fMRI session. The penetration hole that we used is marked in Figure 5*C* (black crosshair), and the trajectory is shown on the rotated coronal and sagittal MRI sections in Figure 5*D*,*E* (orange lines).

### Preparation of the microdrive assembly and its installation

After identifying the desired penetration hole, a microdrilling technique is used to create a minimum opening through the cranium (here, ϕ0.7 mm; Ideal Micro-Drill, Harvard Apparatus; Fig. 6*A*). To start, the acrylic layer within the implant is cleaned. The penetration grid is then cleaned with isopropyl alcohol and installed on the implant. A sterilized drill bit with a drill stop collar is then prepared with a bore depth that is slightly longer (e.g., 1 mm) than the thickness of the penetration grid (here, 5 mm). The top of the penetration grid serves as a reference for determining drilling depths and guide tube lengths. After the initial drilling, the drill bit length is sequentially incremented (e.g., in 1 mm steps) until it passes through the cranium. Before engaging the drill, a subsequent drill bit is first used to hand-verify whether the previous bit passed through the cranium. Specifically, the drill bit is lowered through the penetration grid until one of two possibilities occur. If the bit stops before the drill stop collar contacts the penetration grid, then the previous bit did not pass through the cranium and drilling should continue. If the drill bit lowers until the drill stop collar contacts the penetration grid, then the previous drill bit passed through the cranium and drilling should cease. The bore depth that passed through the cranium is used to determine the guide tube length. The guide tube should be slightly longer than the final drill bit plus the distance from the top of the penetration grid to the top of the stopper. In that case, when the guide tube and microdrive body assembly are installed (described below), the guide tube punctures the dura but minimally impinges on the brain.

For MRI studies, guide tubes should be made of a non-metallic material (e.g., silica tubing). However, such materials may not be rigid enough to puncture the dura. This challenge can be overcome by adding an intermediate step in which the dura is punctured using a sterile hypodermic needle. Specifically, the dura can be punctured by preparing and inserting a sterile hypodermic needle that is slightly longer than the final drill bit. After removing the hypodermic needle, a non-metallic guide tube can pass through the opening made in the dura.

Once a guide tube is prepared, it is soaked in a cleaning solution and secured (e.g., using super glue or other high-strength adhesive) to the hole in the stopper that matches the identified hole in the penetration grid (Fig. 6*B*). The guide tube should be flush with the top of the stopper to ensure the intended guide tube length. The probe is then loaded through the guide tube and secured (again using a high-strength adhesive) to the corresponding hole in the coupler (Fig. 6*B*). To independently control multiple probes, this process can be repeated with multiple actuators (up to four in the presented design). Each actuating mechanism is then loaded into a chamber of the microdrive body, sliding it down until the stopper rests on the stopping notches within the chamber (Figs. 3*B*, 6*C*). The loaded actuators are then secured to the microdrive body using the microdrive cap, which attaches to the top of the microdrive body with four Nylon screws (Figs. 1*C*, 4*A*). The microdrive body assembly is then ready to be installed on the implant (Fig. 6*C*).

Before link installing the microdrive body assembly, four guide rods are screwed into the edges of the implant (Figs. 2*A*,*B*, 6*D*, left). The guide rods are used to align the microdrive body with the implant and penetration grid, ensuring that the guide tubes and probes (which are secured to stoppers and couplers, respectively) maintain alignment with the prepared penetration holes. The aligned microdrive body assembly is then slowly slid down the guide rods toward the implant while monitoring that the tips of the guide tubes enter the appropriate holes in the penetration grid (Fig. 6*D*, middle). Once the microdrive is fully lowered, it is secured to the implant with four Nylon screws (Fig. 6*D*, right). After this setup is complete, a probe can be manually lowered/raised by rotating the actuating rod via the rotating handle. To use the microdrive in an MRI study, the guide rods are unscrewed and removed through the top of the microdrive cap before scanning to prevent imaging artifacts.

If using an electrode, a direct connection to a head stage amplifier can be made with electrical wires. For stereotrodes or tetrodes, an additional electrical interface board can be attached to the top of the microdrive body (not shown here but included with the CAD files).

### Experimental procedures

All procedures were approved by the IACUC at Sungkyunkwan University (SKKUIACUC2019-03-11-1) and were in accordance with the NIH *Guide for the Care and Use of Laboratory Animals*. An adult male rhesus monkey (*Macaca mulatta*; weight, 10 kg; age, 7 years) was implanted with an MRI-compatible round-shaped PEEK headpost (Micro Integration Technology) that was secured using ceramic screws (Thomas Recording) and dental acrylic (Unifast Trad). The microdrive implant was secured to a layer of acrylic over the cranium. A postsurgical structural scan was used to confirm the location of the implant and to estimate the penetration trajectory (Fig. 5).

### MRI scanning preparation

Based on the estimated trajectory (Fig. 5), the penetration grid was used as a guide to drill two holes through the cranium. One hole was for a stimulating electrode and the other was for a ground wire (Fig. 6*A*). Sterile hypodermic needles were then used to puncture corresponding holes in the dura. A stimulating electrode and ground wire were prepared and loaded into fused silica guide tubes (OD: 666 μm, ID: 449 μm; Polymicro) that were secured to separate actuating mechanisms (Fig. 6*B*,*C*). The microdrive body assembly was then installed on the implant and secured using Nylon screws (Fig. 6*D*). During the EM session, no contrast agent was applied to the implant or penetration grid. Finally, the stimulating electrode was lowered into the brain and the ground wire was lowered into the epidural space.

**Figure 7.**
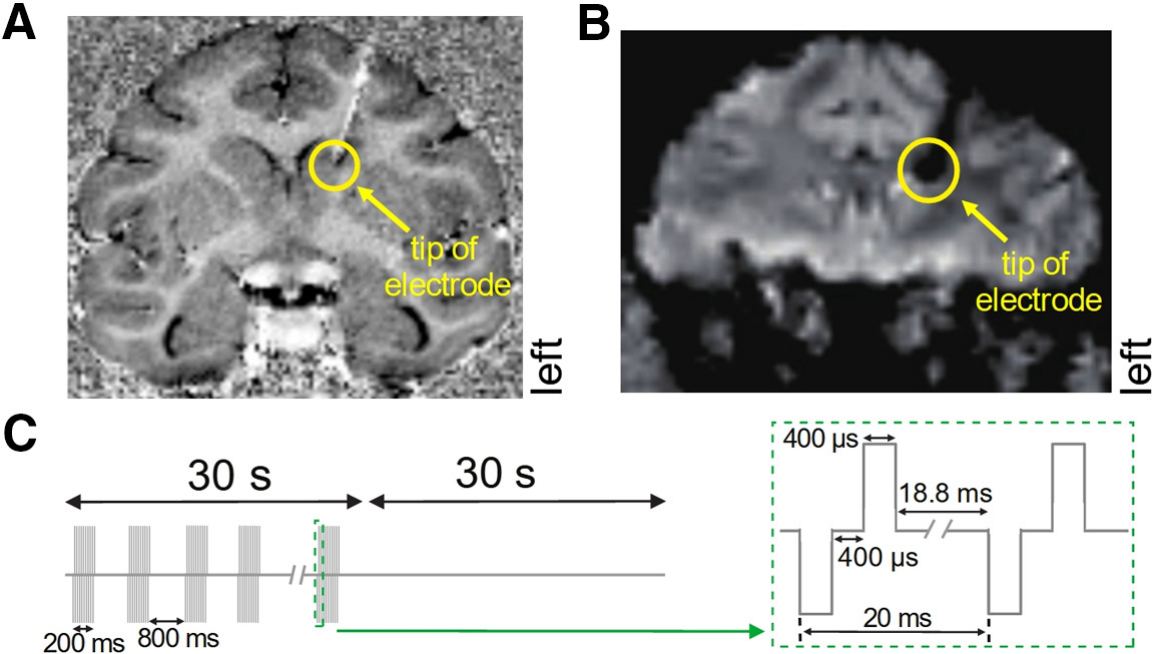
Electrode trajectory and EM schematic. ***A***, Coronal section from a structural scan with the electrode tip in the caudate nucleus. The electrode is visible as a black line and a metal-induced susceptibility artifact (white). The yellow circle marks the location of the electrode tip. Note that the microdrive caused little-to-no image distortion, even directly below the implant (above where the electrode enters the brain). ***B***, Coronal section from a BOLD scan averaged over 540 repetitions with the electrode in place. The electrode (here, a black artifact) and tip (yellow circle) are visible but relatively little distortion from the microdrive is apparent. ***C***, Schematic of the EM schedule. A train of 400 μs square waves (500 μA) at 50 Hz lasting 200 ms was delivered every 1000 ms (800-ms spacing) for 30 s. Periods of stimulation (30 s) and no stimulation (30 s) were interleaved.

MRI images were acquired under anesthesia (isoflurane, ∼1–1.2%). The electrode trajectory was confirmed with structural and blood oxygenation level-dependent (BOLD) imaging (Fig. 7*A*,*B*) before the injection of MIONs (10 mg/kg; total MION = 100 mg; Biopal).

### fMRI scanning

Experiments were conducted using a 7T MRI scanner (Terra, Siemens Healthineers) with a 28-channel knee coil (inner diameter, 15.4 cm). Structural images were acquired using a magnetization-prepared 2 rapid acquisition gradient echoes (MP2RAGE) sequence (TR = 4.3 s; TE = 2.12 ms; slice thickness = 0.5 mm iso). BOLD and MION-enhanced CBV data were collected using a gradient-echo echoplanar imaging (GRE-EPI) sequence (TR = 1.5 s; TE = 20 ms; slice thickness = 0.9 mm iso; 52 slices) with whole-brain coverage.

### Electrodes and EM plan

Two 231 μm diameter Platinum-Iridium electrodes (exposed tip diameter ∼2–3 μm; MicroProbes) were used for stimulation (impedance = 30 kΩ) and grounding (impedance ≤ 500 Ω). Electrical impulses were generated by a stimulator with two isolators (Master-9 and ISO-Flex stimulus isolator, A.M.P.I.) to apply biphasic current pulses. Each EM block was triggered and synchronized with the scanning procedures by TTL signals from the MRI scanner (MATLAB, MathWorks). Stimulation consisted of trains of biphasic cathode-leading currents with a pulse width of 400 μs and a current of 500 μA, repeated at 50 Hz (Fig. 7*C*, right). Stimulation trains lasted 200 ms and were repeated every second for 30 s. Periods of 30 s of stimulation and 30 s of no stimulation were interleaved (Fig. 7*C*, left). Each stimulation session consisted of 13 blocks (i.e., 13 periods of stimulation and 13 periods of no stimulation interleaved).

### Image processing and data analysis

Analyses were performed using MATLAB and the Canlab SPM-based fMRI toolboxes (https://github.com/canlab/CanlabCore; Woo et al., 2017). Structural images were calculated using the following equation:

(1) $$I=\mathrm{Real}\left(\frac{A^{*}\cdot B}{{\left|A\right|}^{2}+{\left|B\right|}^{2}}\right),$$

where A is the first inversion contrast image, $A^{*}$ is the complex conjugate of A, B is the second contrast image from the MP2RAGE sequence, and $\left|\cdot \right|$ denotes absolute value (Marques et al., 2010). This method improves the contrast of the brain, but also increases noise outside of the brain as well as metal-induced susceptibility artifacts (Fig. 7*A*). Structural images were then co-registered to the mean functional images, reoriented to the D99 atlas (Reveley et al., 2017), and segmented into gray and white matter. Functional images were re-oriented using the reorientation matrix obtained from the structural re-orientating process. The images were then motion-corrected, normalized, and smoothed with a Gaussian kernel (1.0 mm full-width at half-maximum). High-pass filtering (cutoff frequency = 0.008 Hz) was used to remove low-frequency signal drifts from the fMRI time series. The CBV signal was then inverted because an increase in blood volume lowers the signal and darkens the image intensity.

Activation maps based on average CBV measurements across stimulation blocks were constructed using a general linear model (GLM) with a design matrix that included a regressor for the EM by convolving the stimulation profile with a boxcar hemodynamic response function. Head movement parameters were accounted for by including linear and quadratic realignment parameters based on current and previous volumes. Statistical maps were then overlaid on the D99 monkey brain atlas to show the areas activated by EM. Voxel-wise t-contrast activations on the spatial maps (false discovery rate, q < 0.05) were used to determine significant activations. The temporal pattern of the CBV response for each stimulation block was constructed by averaging CBV time courses across all voxels within a region of interest (ROI) that had GLM β values >6 (corresponding to the 80th percentile of non-zero β values across all analyzed regions). Voxels belonging to an ROI were determined using the D99 atlas. Activations were reported as percent signal change.

The CAD files and a parts list for the MRI compatible microdrive are available here: https://osf.io/tnpmk/. We encourage others to make modifications based on their specific research needs and hope that our designs facilitate neuroscience research by reducing the time and effort necessary to solve microdrive-related technical issues.

## Results

To test the functionality of the microdrive, we used it to deliver EM to the dorsal caudate nucleus (dCN), ventral caudate nucleus (vCN), and nucleus accumbens (NA) of a male macaque monkey in an EM-coupled 7T fMRI study. Structural and BOLD images were periodically taken while lowering the electrode into these areas to verify the electrode’s location (Fig. 7*A*,*B*). Importantly, we found that the microdrive generated minimal imaging artifacts in the structural scans, even directly below the implant (Fig. 7*A*). Even in the BOLD images, which are more vulnerable to artifacts than structural images (Murakami et al., 2016), there was relatively little image distortion from the microdrive (Fig. 7*B*). The penetration grid was not visible in these images because it was not filled with a contrast agent during the EM session. As expected, the electrode was visible in both the structural and BOLD scans (Fig. 7*A*,*B*, respectively). It appears as a white metal-induced susceptibility artifact in the structural image that is prominent because of how the image was calculated from the MP2RAGE scans (Eq. 1). Importantly, these images confirm that the microdrive itself introduced minimal imaging artifacts, even at 7T, making it possible to monitor the position of the probe with a high degree of accuracy.

**Figure 8.**
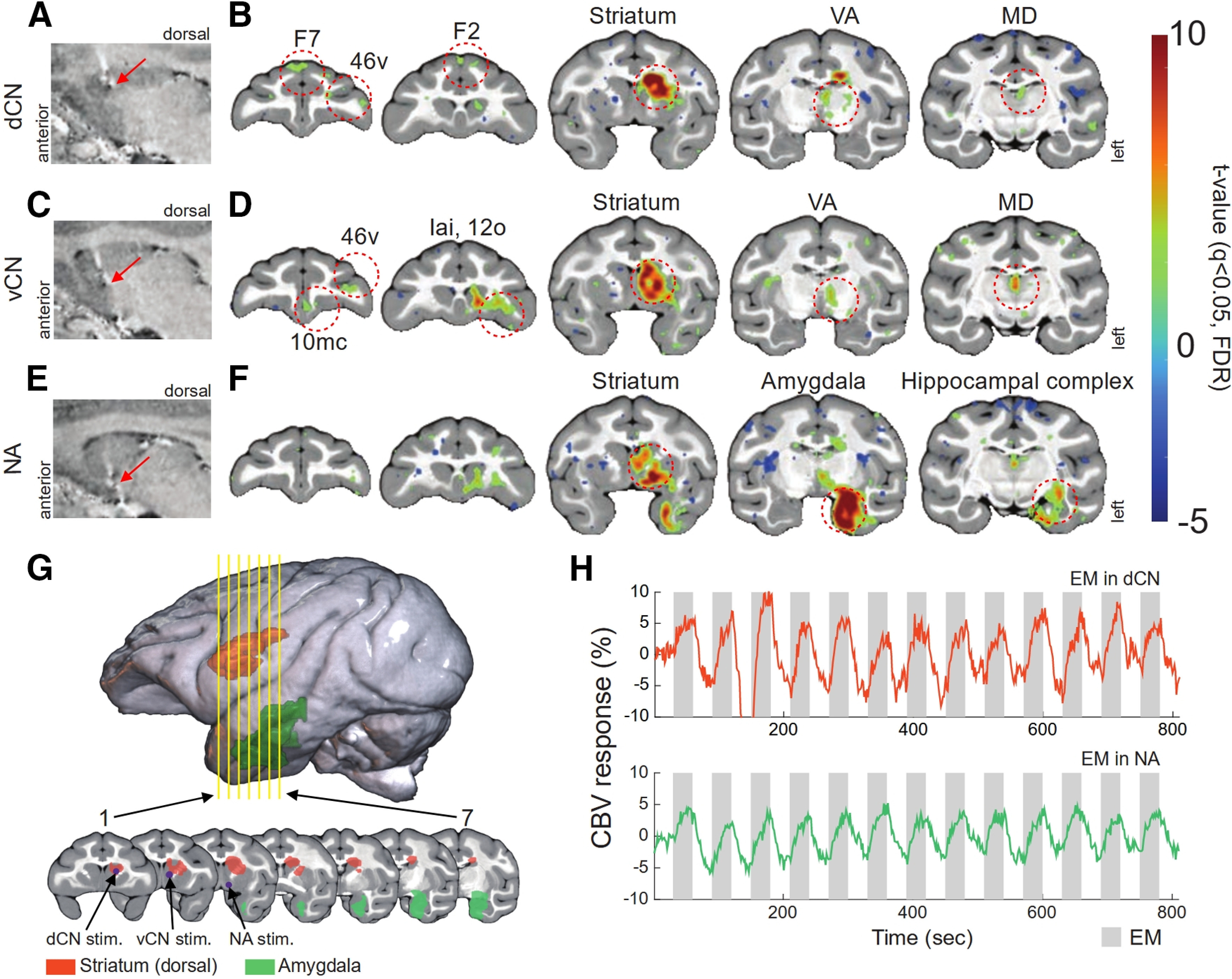
Activation maps and temporal patterns of CBV during EM. ***A***, Location of the first stimulation site (dCN, red arrow) on a sagittal view. ***B***, Activation maps during dCN EM overlaid on the D99 atlas with ROIs outlined and labeled (red circles). ***C***, Location of the second stimulation site (vCN, red arrow) on a sagittal view. ***D***, Activation maps during vCN EM overlaid on the D99 atlas with ROIs outlined and labeled (red circles). ***E***, Location of the third stimulation site (NA, red arrow) on a sagittal view. ***F***, Activation maps during NA EM overlaid on the D99 atlas with ROIs outlined and labeled (red circles). ***G***, 3D rendering of the D99 atlas (top) with two ROIs colored (orange, dorsal striatum; green, amygdala). Seven coronal sections are also shown (bottom; yellow lines from top). On sections 1, 2, and 3, purple dots and black arrows mark the dCN, vCN, and NA stimulation sites, respectively. ***H***, Time course of percent signal change (colored lines) measured in the dorsal striatum during EM in the dCN (top) and amygdala during EM in the NA (bottom). Gray bars indicate 30-s stimulation periods and white bars indicate 30 s no stimulation periods (all 13 blocks are shown). To facilitate comparisons, the time courses are plotted with the same ordinate range. This results in some clipping in the top trace. dCN, dorsal caudate nucleus; vCN, ventral caudate nucleus; NA, nucleus accumbens; F2, agranular frontal area F2; F7, agranular frontal area F7; 12o, orbital prefrontal area; 46v, ventro-lateral prefrontal area; 10mc, medial prefrontal area; Iai, intermediate agranular insula area; MD, mediodorsal thalamus; VA, ventral anterior thalamus.

To assess the effects of EM delivered using the microdrive, we applied EM at three locations in the left hemisphere: the dCN, vCN, and NA. At each location, 13 blocks of EM were delivered. The resulting activation maps are shown in Figure 8. We first stimulated the dCN (penetration depth from the dura = 13.14 mm; Fig. 8*A*, red arrow) and found that activity significantly increased in the ipsilateral striatum, agranular frontal area F2, ventro-lateral prefrontal area (46v), mediodorsal thalamus (MD), and ventral anterior thalamus (VA; Fig. 8*B*). A significant increase in activity was also observed in the contralateral agranular frontal area F7. In the same imaging session, we lowered the stimulating electrode into the vCN (penetration depth from the dura = 18.57 mm; Fig. 8*C*, red arrow). During EM of the vCN, we found that activity significantly increased in the ipsilateral striatum, 46v, MD, VA, medial prefrontal area (10mc), intermediate agranular insula area (lai), and orbital prefrontal area (12o; Fig. 8*D*). The observed activations associated with dCN and vCN EM were consistent with the known anatomic connectivity of the striatum (McFarland and Haber, 2002; Haber, 2016). Lastly, we lowered the stimulating electrode into the NA (penetration depth from the dura = 22.88 mm; Fig. 8*E*, red arrow). During EM of the NA, we found that activity significantly increased in the ipsilateral striatum, amygdala, and hippocampal complex (Fig. 8*F*). These observed activations were consistent with the known anatomic connectivity of the NA (Alexander et al., 1986; Friedman et al., 2002; Choi et al., 2017).

To confirm that the temporal pattern of the CBV responses followed the EM schedule, we calculated the time course of activation in select ROIs during dCN and NA stimulation. For stimulation of the dCN, we calculated the activity within the dorsal striatum, which included the stimulation site (Fig. 8*G*,*H*, orange). For stimulation of the NA, we calculated the activity within the relatively distal amygdala (Fig. 8*G*,*H*, green). In both cases, the pattern of activity was robust and temporally locked to the EM schedule, with the signal increasing after the onset of stimulation (Fig. 8*H*, gray bars) and decreasing after stimulation ceased (Fig. 8*H*, white bars). These results confirm that the MRI compatible microdrive could be used to reliably deliver EM to designated brain areas in a 7T MRI environment with minimal imaging artifacts.

## Discussion

For many neuroscience experiments, the microdrive is an essential nexus between neuronal activity and data acquisition. To satisfy the specific demands of a study, it is often necessary to customize the microdrive’s design to ensure accurate and reliable control of probes. Such customization introduces additional development time and manufacturing costs, particularly when it relies on conventional machining. A potentially more effective approach to customizing microdrives is to use 3D printing capabilities with sub-millimeter resolution. Indeed, the utility of 3D printing has recently become apparent across a wide range of research and medical applications (Liaw and Guvendiren, 2017; Jamróz et al., 2018; Nagarajan et al., 2018).

Here, we demonstrated the feasibility of using a 3D**-**printed, MRI compatible microdrive in an EM-coupled fMRI study. We found that the workflow from design to CAD to fabrication with 3D printing was seamless, and that 3D printing was especially efficient for constructing the microdrive’s small components. This advantage can further expedite customization. For example, the presented design includes four chambers to accommodate four independent actuators, but the supplied CAD files can be easily modified to print a microdrive body that houses fewer or more actuators depending on the number of target areas and the brain size. The length of the chamber can also be easily modified to accommodate shorter or longer travel distances. Importantly, the simplicity of the workflow can support an almost immediate response to new experimental demands, which is not always possible with commercial manufacturers.

### Microdrilling reduces imaging artifacts and implant maintenance

The microdrive produced no substantial imaging artifacts because it was made of MRI compatible materials and few metallic parts. In addition, the use of a penetration grid and microdrilling technique eliminated the need for a larger craniotomy and conventional chamber which can create an air-filled space that is problematic for imaging and can potentially result in deformation of the underlying neural tissue. To minimize damage to the dura and neural tissue during microdrilling, a drill stop collar was used to ensure precise control of the bore depth. By hand-verifying the bore depth, it is possible to determine the drill bit length that passes through the cranium within the tolerance of the step size that the drill bits are incremented (e.g., 1 mm). We have used variations of this technique for years (Rosenberg et al., 2013; Laurens et al., 2016; Chang et al., 2020), and have not encountered problems with infection. Meticulous cleaning of the acrylic layer before and after drilling, cleaning and sterilizing components, and covering the implant outside of the experiment (CAD files for a cover are provided but not shown) greatly reduce the risk of infection. However, if infection occurs, the local acrylic layer could be removed and the infection treated topically. The acrylic layer could then be replaced. Thus, the microdrilling technique helps reduce imaging artifacts associated with larger craniotomies as well as implant maintenance.

### Performance of the microdrive during EM-coupled MRI scanning

The microdrive showed excellent performance in a 7T MRI environment where it was used to position a stimulating electrode and deliver EM. We placed the electrode in the dCN, vCN, and NA, and delivered EM during fMRI scanning to assess the effective connectivity of these areas across the brain. For each stimulation location, we found that the induced activation was largely restricted to the ipsilateral side of the brain, consistent with some previous findings (Tolias et al., 2005; Matsui et al., 2011). However, it is also possible that our imaging methods limited the ability to detect contralateral activations. Indeed, other studies have shown some contralateral activations induced by EM, consistent with anatomic connections across hemispheres (Ekstrom et al., 2008; Moeller et al., 2008; Premereur et al., 2015; Murris et al., 2020). As expected for the EM of striatal regions, the activated areas were known constituents of the basal ganglia circuit (Draganski et al., 2008; Haber and Knutson, 2010). The results thus confirmed that our MRI compatible and 3D**-**printed microdrive can be used to examine the connectivity of brain networks using EM-coupled whole-brain imaging.

### Future applications and current limitations

Here, we demonstrated the feasibility of using our 3D**-**printed microdrive in an EM-coupled fMRI study. Because of the versatile design of the actuating mechanism, the microdrive can accommodate various types of electrodes for neuronal recordings (e.g., linear arrays, stereotrodes, or tetrodes), cannulae for inactivation or neuropharmacological studies, or optogenetic fibers for precise neuronal modulation. Thus, the microdrive can support a wide range of future applications. In the current design, a probe is advanced by manually rotating an actuating rod. However, studies often require a period in which the probe must be moved frequently while continuously monitoring neuronal activity (e.g., while approaching a target brain area). In such cases, a motorized actuating module would be more efficient than the current manual mechanism. We therefore have plans to incorporate a motorized actuating module with control software as an extension of the existing design. Lastly, our skull-mounted frameless design is potentially suitable as a chronic microdrive for longer-term applications. The future addition of a protective housing could enable the microdrive body to stay attached to the implant for an extended period of time, potentially with freely moving animals (Wilson et al., 2003; Greenberg and Wilson, 2004; Sun et al., 2006).

## Synthesis

Reviewing Editor: Leonard Maler, University of Ottawa Decisions are customarily a result of the Reviewing Editor and the peer reviewers coming together and discussing their recommendations until a consensus is reached. When revisions are invited, a fact-based synthesis statement explaining their decision and outlining what is needed to prepare a revision will be listed below. The following reviewer(s) agreed to reveal their identity: Li Chen.

Editorial comments

Both reviewers agree that this manuscript provides a detailed description of what is likely to be an important tool for advanced neuroscience research. Neither reviewer or the Editor has found any serious difficulties with any aspect of this manuscript. The reviewers did identify a number of minor issues that need to be addressed but the revisions will only require an Editorial review. Below I have appended the pertinent comments of the reviewers.

Please be sure to provide a concise rebuttal and a redlined version of the Ms to facilitate the Editors final quality control check of the manuscript and congratulations on your important and timely contribution.

Reviewer comments.

The authors of this manuscript provide a detailed description of a 3D printable microdrive, which is MR compatible. The CAD drawings will also become available upon publication of the manuscript and are supposed to be adaptable to the specific needs of a researcher (e.g. the number of actuators can be changed, etc.). Since the combination of whole brain functional imaging and electrical microstimulation is becoming a more popular research tool in systems neuroscience to assess ‘effective connectivity’, I highly commend the authors for these developments and especially for sharing them with the community. As indicated by the authors, the device could be easily adapted to accommodate gear needed for focal pharmacological, optogenetic and other types of interventional studies. Hence, the expected impact may be quite large.

The paper is well written and easy to digest. Of course, the proof of the pudding is in the eating. It is difficult to assess how easy it will be to construct the MR compatible Microdrive itself based on the description without having access to the designs themselves. I fully trust that this should be reasonably easy, however. Altogether, I’m very enthusiastic about this manuscript and about the exceedingly useful CAD designs that will come with it. I only have a few minor remarks and suggestions, which may improve the manuscript when they are considered.

1. Regarding he micro-drilling: of course, this procedure has several benefits as pointed out by the authors (reduced imaging artifacts, reduced maintenance typically associated with craniotomies, etc.). However, it is not trivial to have control over the depth of the drilling (how do you know that you drilled through the cranium without touching the dura?) ; to avoid larger blood vessels when inserting the guide tube or electrode (which is easier to avoid when using a larger craniotomy); detect and control small infections in and under the burr holes; etc. It would be helpful if the authors can be more specific as how to make the small holes and how to deal with issues listed above?

2. In the introduction it may be worthwhile to point to a few existing MR compatible microdrives that have been used before -e.g. by the Leopold and Tsao laboratories (e.g. McMahon et al. PNAS 2014; Moeller et al., Science 2008).

3. Line 184: the purpose of the elastic tube is unclear. Doesn’t it induce unwanted/uncontrollable vertical motion (e.g. sudden jumps in the Z-direction)?

4. A slightly more detailed description as to how the probes (e.g. electrodes) are fixed would be useful. It doesn’t seem to be similar to a fixation procedure using standard microdrives.

5. The fiducial markers (line 230) (i.e. the filled grid) seem to be too short to accurately estimate the trajectories. A small angular deviation may result in a huge error, certainly when targeting deeper structures. I would suggest that using a longer filled MR compatible guide tube may be more optimal for targeting deep structures (e.g. as used in the peri-operative targeting procedures described in Arsenault et al., Current Biology 2014).

6. Line 280: a more detailed description (or figure) of the stopper and coupler may be useful.

7. Line 332: I’m surprised by the white artifact from the electrode. Isn’t this contrast agent leaking from the grid along the trajectory? Also, I’m surprised by the quality of the activation maps and the timecourse of the CBV response d(Fig 8) despite the rather low quality of the EPIs -probably due to the use of a non-optimal knee coil? (Fig 7B). I may have missed it, but how much data were acquired (and actually used) to generate the statistical maps and timecourses? How much MION was injected?

8. The electrodes are also very thick. What is the reason? I’m surprised that the impedance for such thick electrodes is not lower than 30 kOhm. Was the (exposed) electrode tip much thinner?

9. Timecourses in Figure 8 were sampled in the head of the caudate. What was the distance between the electrode tips and the ROI where the timecourse was sampled? Was this the average timecourse of all voxels in the head of the caudate nucleus, or was it derived from just the peak voxel? It would be interesting to show timecourses from a few other sites that showed elevated EM-induced fMRI activity.

10. Line 438: "the 3D printing was highly efficient in terms of the design time and manufacturing costs." I may have missed it, but how long was the time and what was the manufacturing cost?

11. Line 458: "induced activation was largely restricted to the ipsilateral side of the brain consistent with previous findings (Tolias 2005; Matsui 2011)." This statement is not entirely correct. The Tolias study was performed with a local coil, so that approach was not really representative for assessing laterality effects. In fact, several other studies, including the first "whole" brain EM-fMRI studies by Ekstrom et al., Science 2008 and Moeller et al., Science 2008; ( and later also by e.g. Premereur et al. PLos Biology 2015; Muris et al., Cer Cortex 2020) showed contralateral activations induced by EM, which is also consistent with (weak) anatomical connections across hemispheres. In fact, there are contralateral striatial-cortical and cortico-striatal connections which should be picked up by EM-fMRI. So maybe the measurements were not sensitive enough? This should be acknowledged.
